# Measuring collagen injury depth for burn severity determination using polarization sensitive optical coherence tomography

**DOI:** 10.1038/s41598-022-14326-3

**Published:** 2022-06-21

**Authors:** Taylor M. Cannon, Néstor Uribe-Patarroyo, Martin Villiger, Brett E. Bouma

**Affiliations:** 1 Institute for Medical Engineering and Science, Massachusetts Institute of Technology, Cambridge, MA 02142 USA; 2 Wellman Center for Photomedicine, Massachusetts General Hospital, Boston, MA 02114 USA

**Keywords:** Biomedical engineering, Biophysics, Medical research, Optics and photonics, Optical physics, Electrical and electronic engineering

## Abstract

Determining the optimal treatment course for a dermatologic burn wound requires knowledge of the wound’s severity, as quantified by the depth of thermal damage. In current clinical practice, burn depth is inferred based exclusively on superficial visual assessment, a method which is subject to substantial error rates in the classification of partial thickness (second degree) burns. Here, we present methods for direct, quantitative determination of the depth extent of injury to the dermal collagen matrix using polarization-sensitive optical coherence tomography (PS-OCT). By visualizing the depth-dependence of the degree of polarization of light in the tissue, rather than cumulative retardation, we enable direct and volumetric assessment of local collagen status. We further augment our PS-OCT measurements by visualizing adnexal structures such as hair follicles to relay overall dermal viability in the wounded region. Our methods, which we have validated ex vivo with matched histology, offer an information-rich tool for precise interrogation of burn wound severity and healing potential in both research and clinical settings.

## Introduction

There is a need for tools to accurately evaluate the absolute depth extent of burn injuries in skin. Dermatological burns are a common injury, accounting for nearly 500,000 annual emergency department visits in the United States^1^. Although the severity of burn wounds is defined by how deeply the tissue is affected, burns are generally clinically assessed only at their surfaces by eye or camera. Among expert clinicians, this approach is effective in identifying mild, first-degree burns limited to the epidermis (the uppermost layer of the skin), as well as severe third-degree burns that extend through the dermis (the second layer of skin) to underlying subcutaneous fat or bone. However, even expert burn wound clinicians face an inaccuracy rate of 25-40% when evaluating second-degree, or partial thickness, burns, which encompass a wide range of injuries with some penetration into the dermis^2^. This presents a clinical challenge in proper management of burn wounds: shallower partial-thickness burns are expected to behave more similarly to first-degree burns and require minimal intervention, whereas deeper partial-thickness burns, analogously to third-degree burns, may require grafting for proper healing^3^. Partial-thickness burn wounds would also benefit from longitudinal monitoring; improperly treated burns may deepen over time, whereas properly managed burns see the healthy (re-)generation of scar and dermal tissue^4^. Therefore, there is strong clinical motivation for a non-invasive tool for longitudinal monitoring of burn wound injury depth to inform optimal treatment for patients.

The gold standard for quantitatively evaluating burn wound severity is punch biopsy followed by histological evaluation^5^. Not only does tissue biopsy lead to additional scarring and potential disruption of the healing process, but burn severity can vary greatly over even a very small area of tissue, complicating prediction of clinical outcome. Histological evaluation of burn wounds that extend into the dermis reveals the denaturation of dermal collagen, giving hematoxylin and eosin (H&E)-stained collagen fibers a glassy or “gelatinized” appearance^6^. Given the lack of tools for comprehensive collagen evaluation, it remains a challenge to understand how collagen damage directly relates to patient outcomes. Therefore, a key supplementary evaluation is identification of adnexal structures, such as hair follicles, which harbor stem cell reservoirs and drive dermatological healing^7^. Beyond evaluation of burn severity as first, second, or third degree, the presence of damaged or intact follicles at a given site informs healing potential, yielding valuable clinical prognostic information. However, with current gold-standard methods, the evaluation of these structures is limited to their incidence in histological cross-sections, which only sparsely sample the tissue compared to the full burn volume.

To more comprehensively characterize full burn volumes, various imaging approaches have been explored for burn wound assessment. Conventional medical imaging modalities such as MRI and ultrasound lack sufficient resolution to finely distinguish burn injury depths on the relevant scale ( tens of microns). As an alternative, optical imaging technologies offer higher resolution and a unique opportunity to probe the integrity of the dermal collagen matrix based on the interaction between this layer and the polarization of light. In healthy skin, the dense collagen matrix of the dermis exhibits high birefringence: collagen’s fibrillar structure strongly modulates the polarization state of incident light^8^. In thermally-damaged, gelatinized regions, collagen fibers denature and fuse, greatly reducing birefringence and polarization transformation^9^. An optical imaging modality which ideally capitalizes on this mechanism for volumetric burn detection is polarization-sensitive optical coherence tomography (PS-OCT), a functional extension of OCT^10^, which cross-sectionally images scattering media, including tissues in vivo and ex vivo, based on intrinsic contrast^11,12^. PS-OCT adds sensitivity to birefringent structures, such as healthy collagen fibers^13^. The volumetric, label-free imaging capabilities of OCT have further advantages over other optical methods, which may fundamentally lack depth-resolved information^14^, require tissue contact^15^, or have insufficent depth penetration to probe the full dermis^16^. In contrast, PS-OCT is capable of non-contact, depth-resolved imaging of tissue to depths up to 2 mm^17^.

Prior work has specifically evaluated the efficacy of PS-OCT in determining burn wound severity^12,18–21^. However, much of this work was limited to measuring cumulative retardation. While OCT is inherently a depth-resolved imaging technique, this metric only relates polarization-based information that is representative of a round trip path of light into and out of the tissue, rather than true characterization of local tissue properties. More recent progress in PS-OCT has enabled the measurement of local retardation (i.e., birefringence) throughout tissue volumes, as well as the degree of polarization (DOP) at each imaged location^22^. DOP is an additional metric accessible via PS-OCT that has been shown to quantify the uniformity of the polarization states, impacted by the underlying tissue birefringence. Longitudinal assessment of DOP has been previously performed to distinguish mature from newly deposited collagen in the context of dermatologic wound healing^23,24^. In this work, we leverage the information yielded by DOP to assess the absolute depth extent of collagen damage as a function of burn wound severity in samples of porcine skin ex vivo, which is a closer structural and immunological match to human skin than that of other commonly used animal models such as rodents, though still suffers from functional limitations such as regenerative capacity^25^. DOP is an advantageous parameter for evaluation because it is measurable using simpler PS-OCT hardware and post-processing algorithms than are required to measure local retardation, bolstering its potential for rapid clinical adoption. Supported by rigorous histological evaluation, we find that DOP is a strong predictor of collagen status in tissues ex vivo, corresponding to burns from superficial to deep partial thickness. We further combine this polarimetric information relating dermal collagen integrity with the visualization of hair follicles to map adnexal structure location, jointly probing thermal injury depth and local tissue regeneration potential. Although thorough correlation between collagen damage and clinical burn outcomes remains yet unresolved, we hope that together, these data will provide a fuller clinical picture of damage and healing potential than existing imaging technologies at a far higher sampling rate than gold-standard punch biopsy methods. Further, our non-invasive, label-free technique potentiates accurate longitudinal evaluation of burn wound severity in vivo to assess the progression of healing over time. We envision this platform will offer value not only in the clinic, but also in research settings, for the evaluation of novel therapies for burn wound treatment and a thorough investigation of the relationship between collagen damage and dermal regenerative capacity that will enhance the translational value of this work.

## Results

**Figure 1:**
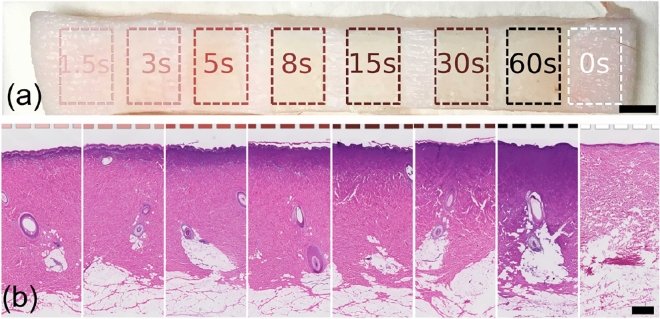
Visual overview of superficial appearance of induced burn wounds by eye (**a**) and upon evaluation with hematoxylin and eosin (H&E) histology (**b**). The colors of the dashed lines here and in subsequent figures correspond to the duration of burn for a given tissue region. Scale bars = 0.5 mm.

**Figure 2:**
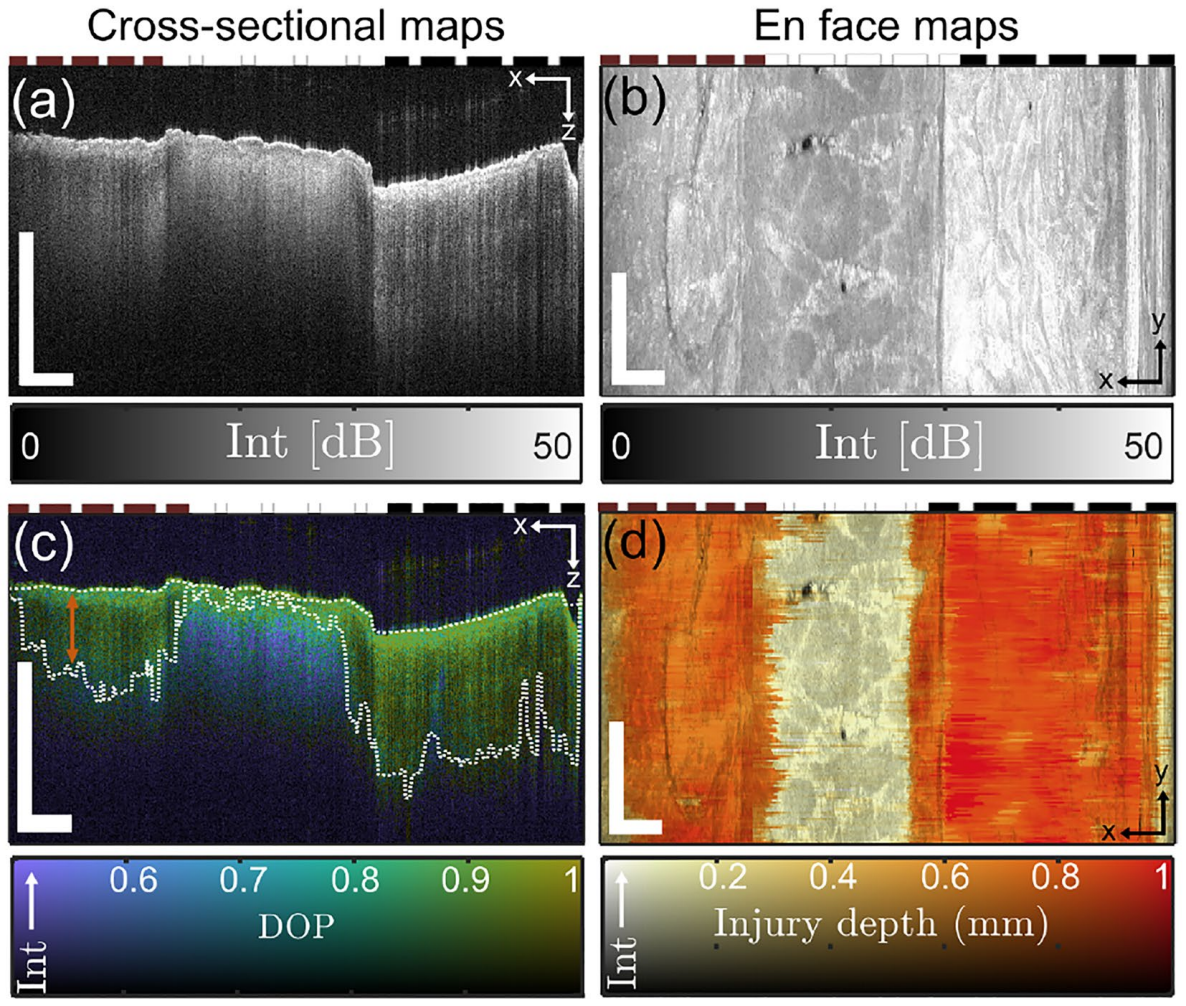
Porcine skin samples were imaged with a PS-OCT system after burn induction. OCT intensity images are shown for a representative cross-sectional view (**a**) and superficial *en face* view (**b**) for an ROI spanning a tissue region subject to 30 s burn duration (left side of each panel), no burn (center of each panel), and 60 s burn duration (right side of each panel). The degree of polarization (DOP) was spatially mapped throughout each cross-section (**c**). The surface of each tissue section was identified automatically and a DOP threshold was applied to determine the depth extent of the injured collagen in each cross section (white dashed lines). The difference between these two lines [e.g., orange arrow in (**c**)], adjusted by tissue refractive index, was used to calculate the collagen injury depth for each cross section of the imaged volume to produce an *en face* map of DOP-based collagen injury depth (**d**) corresponding to the same ROI as (**b**). Scale bars = 1 mm. Intensity limits for 2D colorbars in (**c**) and (**d**) range from 0 dB to 50 dB.

To evaluate DOP as a predictor of dermal collagen damage depth extent and a surrogate measurement of burn wound severity, we induced burns with a continuously heated metal block at a range of durations, corresponding to differing severities, in samples of excised porcine skin [Fig. 1a]. Porcine skin samples were acquired from two different sources (referred to as Datasets 1 and 2), and the increasing burn severity with injury induction time was confirmed histologically [Fig. 1b]. Following burn induction, samples were imaged with a custom PS-OCT imaging system, capturing a volume with surface area of 1.2 cm $\times$ 0.3 cm through a representative portion of each burn wound, as well as undamaged regions [Fig. 2a,b]. Our custom signal and image processing pipeline was used to calculate the DOP for each cross section [Fig. 2c], and based on a given DOP threshold value, estimate the depth-extent of collagen denaturation. Injury depth was calculated for each cross-section and mapped* en face* throughout each volume [Fig. 2d]. We confirmed that while intensity cross-sections and *en face* maps yielded little structural information with clear relevance to thermal damage, higher DOP was observed at increasingly greater depths into tissue with increasing burn duration. This confirmed our expectation that in healthy (unburned) skin, DOP decreases quickly with depth upon reaching the dense matrix of randomly-oriented collagen fibers of the dermis, whereas with loss of birefringent collagen following thermal injury, the polarization properties of incident light are unaltered in superficial burn regions. The resulting DOP remained high until interaction with the healthy, intact collagen matrix below, creating a clear injury border.

**Figure 3:**
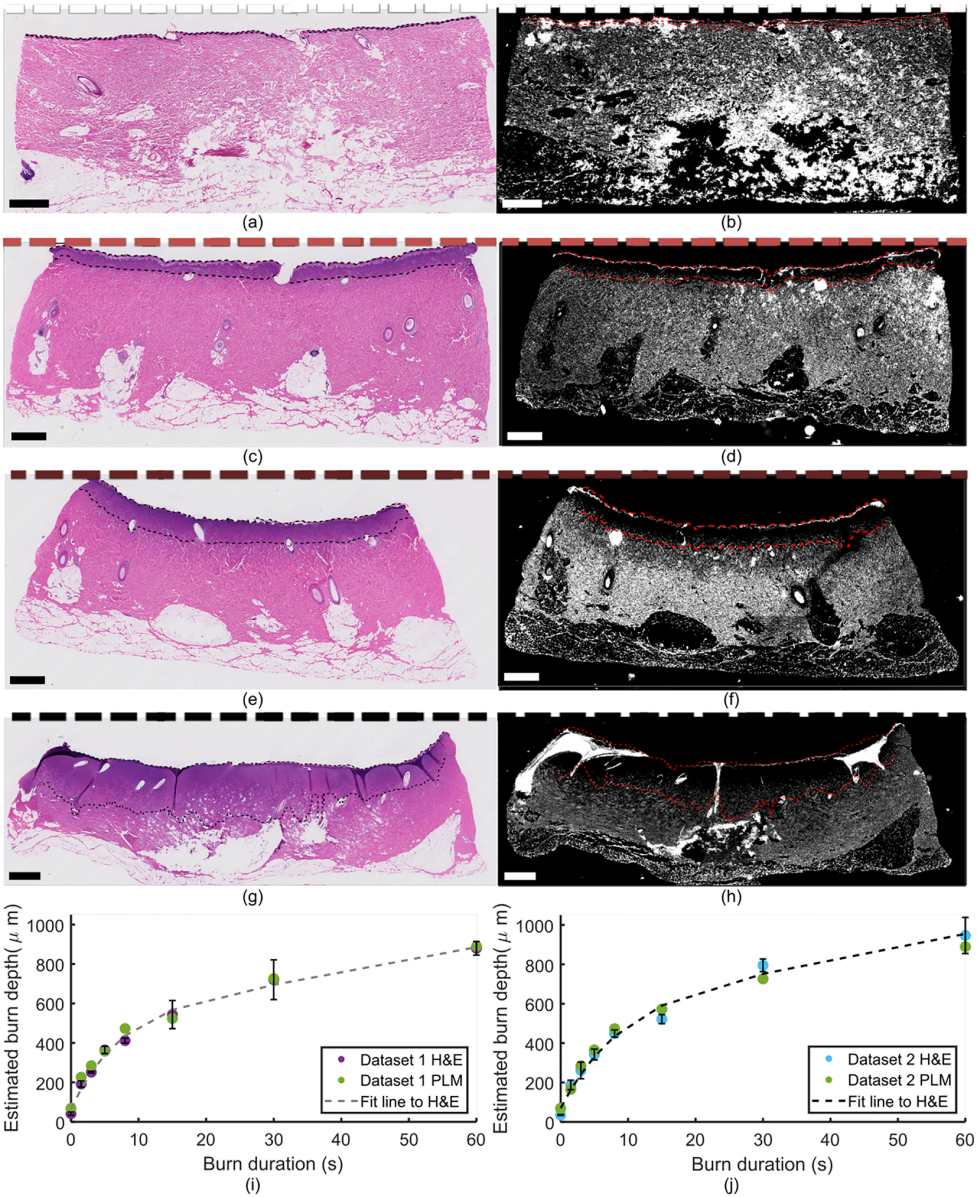
Zones of gelatinization identified with H&E histology for healthy tissue (**a**), and 5s (**c**), 15s (**e**), and 60s (**g**) burns, and corresponding PLM data (**b**, **d**, **f**, **h**). Dashed lines in black (**a**, **c**, **e**, **g**) and red (**b**, **d**, **f**, **h**) show automatically-extracted surface and injury boundaries. Summary of results for Datasets 1 (**i**) and 2 (**j**) show good agreement between H&E and PLM data. Error bars represent standard deviation across N = 6 histological cross sections for each Dataset. Scale bars = 1 mm.

Following imaging, tissue was fixed in 10% formalin and paraffin embedded. Standard histologic sections were processed with hematoxylin and eosin (H&E) staining [Fig. 3a,c,e,g]. Unstained tissue sections were also collected at neighboring locations for polarized light microscopy (PLM) evaluation [Fig. 3b,d,f,h]. In H&E cross-sections, visible zones of gelatinization were consistent with increasing depth of damage at increasing burn durations. Like PS-OCT, PLM is sensitive to collagen and fibrillar structures; in normal dermis, birefringent collagen has a high signal (here, shown as white), and in thermally denatured areas, this contrast is absent (dark). To automatically assess burn injury depths without introducing reader bias, images of H&E sections were analyzed with thresholding in Red-Green-Blue (RGB) channels to define the boundary of the gelatinization zone. Matching PLM sections were analyzed with an applied binary threshold to define the boundary of intact collagen. Together, these results were used as a simple and intuitive ground truth against which to compare the PS-OCT-based burn injury depth estimates. Injury depths estimated at a given threshold value were in good agreement between H&E and PLM assessment for both Datasets and increased monotonically with burn duration [Fig. 3i,j]. The rate of increase, when fit with a double-exponential curve, is similar in shape to previous reports of gelatinization depth and theoretically predicted Arrhenius damage integral combined with a finite element model^6^.

**Figure 4:**
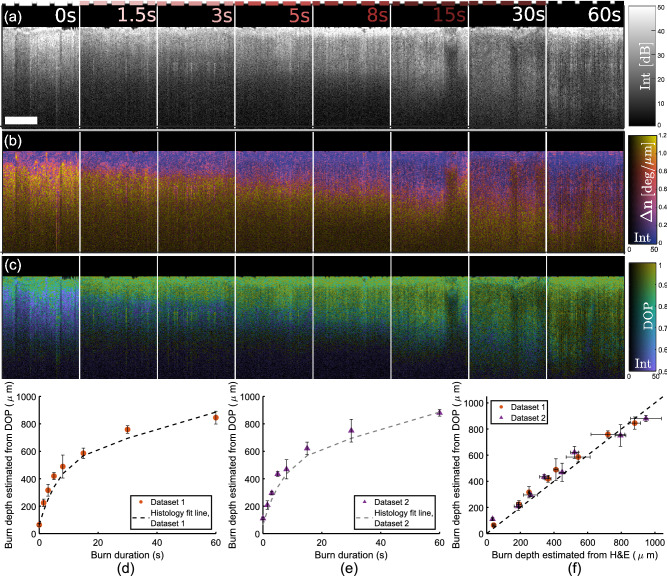
A representative cross-sectional OCT intensity image (**a**), birefringence map (**b**), and DOP map (**c**) is shown for a representative tissue location for each induced burn duration. DOP-based burn depth estimation results, averaged throughout each burned tissue volume, are plotted against histology results from Fig. 3 for Dataset 1 (**d**) and Dataset 2 (**e**). Histology fit line is reproduced from Fig. 3i,j. DOP-based and histology-based burn depth estimates are compared for each Dataset (**f**). Scale bars = 0.5 mm.

Representative cross sections showing OCT intensity [Fig. 4a], tissue birefringence [Fig.4b], and DOP [Fig. 4c] are shown for each burn injury duration. In post-processing, tissue surfaces were flattened for clearer visualization of the relative depth of thermal injury in each cross section. With a DOP threshold set to 0.95, our DOP-based burn injury depths and histologically-determined injury depths agreed well for both Datasets [Fig. 4d,e]. Datapoints were averaged across each of the three volumetric DOP maps for each Dataset. Histologically-determined injury depths from Fig. 3 were fitted to a logarithmic curve for clearer visualization against the DOP-based results. We note that in healthy tissue, the nonzero depth reported for wound injury is due to the lack of birefringence in the epidermis, which causes it to be classified using our methods as a thermally-damaged region. However, given the shallowness of this layer ($\sim$ 50 $\upmu$m), this small discrepancy is not of realistic diagnostic importance for partial thickness burns at many dermatological sites. Induction and measurement of burn depths, using histology and DOP, was also highly consistent between each Dataset [Fig. 4f]. Using a linear regression model, the coefficients of determination for Datasets 1 and 2 were $R^2=0.97$ and $R^2=0.93$, respectively. Burn depths estimated from tissue birefringence were also quantified for both Datasets and results were found to be similar to those estimated from DOP prior to SNR correction, but less accurate than those estimated from DOP post-SNR correction (Supplementary Fig. S1).

**Figure 5:**
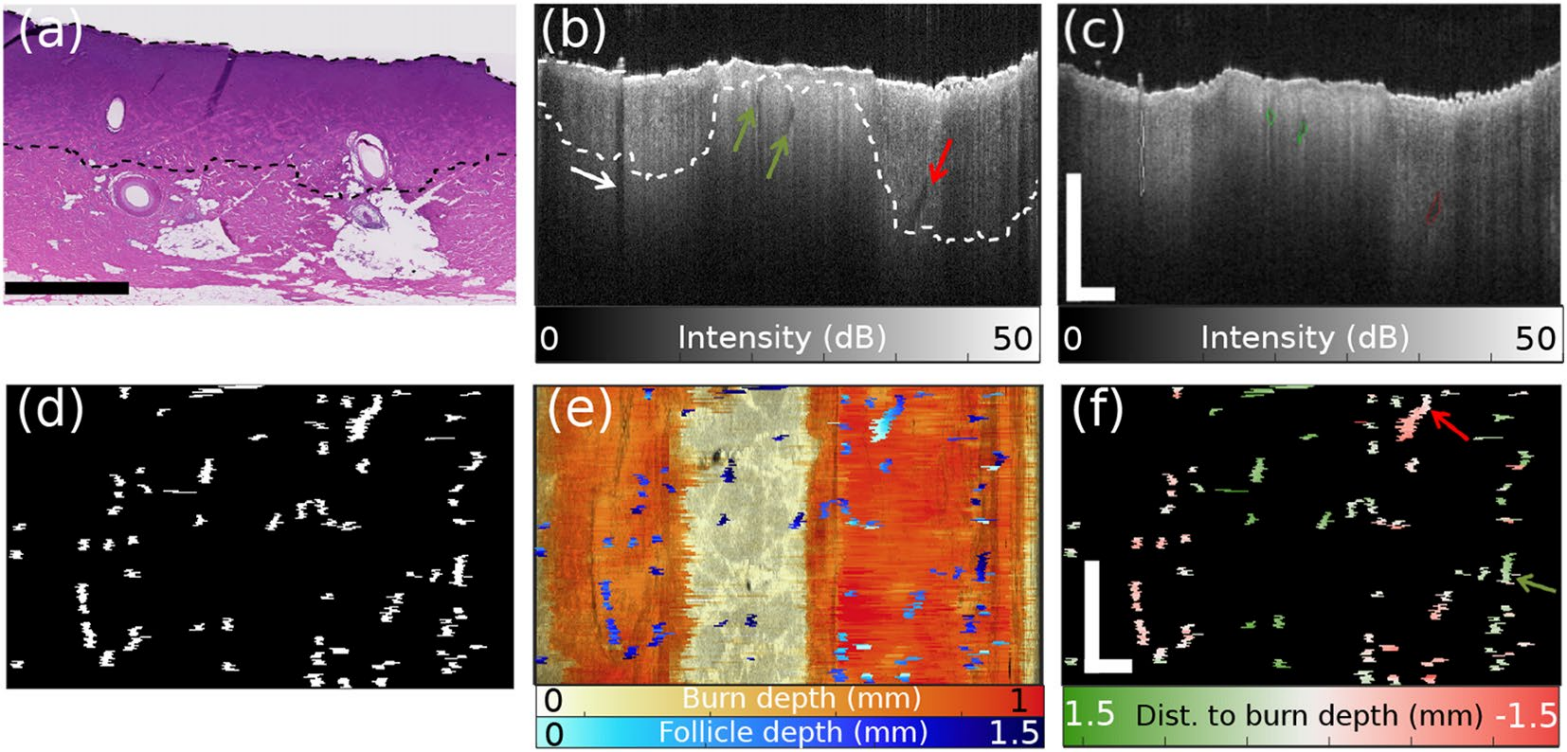
Histological (**a**) examples of mature follicles below (left) and above (right) our DOP-based, automatically-determined collagen damage boundary. In (**b**), we provide examples of hair shaft shadow (white arrow), intact superficial hair shaft cross section (green arrows), and deep follicle within burn zone (red arrow), and the automatic detection of these structures using our image processing routine (**c**), with the shadow being excluded from further processing. Maps were generated for *en face* visualization of the locations of identified follicles (**d**). In (**e**), the depths of those follicles below the tissue surface are encoded against the DOP-based burn depth estimation (see Fig. 2). Our healing potential map directly compares these two parameters as $\text{Distance (Dist.) to burn depth} = \text{depth}_\text{follicle} - \text{depth}_\text{burn}$, where follicles above the border of collagen injury (displayed in green) would be presumed undamaged, and follicles below this border (displayed in red) would be presumed at risk for damage.

Throughout histological cross sections [Fig. 5a] as well as volumetric OCT data [Fig. 5b], many cross-sections of hair shafts and their terminal hair follicles were visible. The content of hair follicles in porcine skin has been previously reported to be similar to that of human skin (though hair follicles in porcine skin may penetrate into the subcutaneous fat, whereas human hair follicles are limited to the dermis)^26^. In general, deep, mature follicles give rise to hair shafts that extend up to the tissue surface. Cross-sections of hair follicles and hair shafts may appear more circular or oblique in geometry depending on their angle with respect to the plane of OCT imaging or histological embedding. With OCT imaging, the close analysis of such cross-sections shows the evolution of these structures with depth throughout a volume. Using a customized image processing routine, follicles were automatically identified throughout an OCT volume featuring regions of moderate and deep burn injury, as well as unburnt tissue [Fig. 5b–d]. The locations of hair follicles, as well as their depthwise location relative to tissue surface, were mapped within the context of DOP-based collagen damage assessment [Fig.  5e]. Additionally, follicular structure depths were directly compared to DOP-based burn injury depth estimation [Fig. 5f] to generate a “healing potential” map: follicle structures located safely below (green, in map) the burned region are more likely to succeed in initiating wound healing than those located near or within the zone of collagen damage (red, in map). Throughout representative histology, we have observed that in general, follicles situated in areas of damaged collagen also exhibit signs of physical damage (Supplementary Fig. S2), based on conventional pathological criteria, such as cell swelling and hyperchromatic nuclei^27^. This suggests that follicles in zones of significant collagen damage are also damaged themselves, an observation supported in existing literature^28^, making them unlikely to effectively contribute to the wound healing process through stem cell-based tissue regeneration. We envision that such *en face* healing potential maps will provide additional insight into both the location and the status of these regenerative structures. Importantly, these maps of healing potential offer more clinical context and prediction of patient outcomes than collagen injury mapping alone.

## Discussion

PS-OCT measurements of DOP in skin show great promise for absolute determination of injury depth in burn wounds based on thermal damage to collagen. Volumetric, longitudinal mapping of collagen matrix integrity would not only fulfill an unmet clinical need where no tools currently exist, but would also provide a valuable tool to researchers investigating new therapies for debridement and accelerated wound healing strategies in animal models of burn injuries. While evaluating novel wound healing therapies, taking punch biopsies can add additional inflammation and alter the tissue microenvironment^5^, prioritizing the development of precise yet noninvasive methods. Our approach is straightforward and realizable with commercial OCT systems with polarization-diverse detection; since DOP measurements only require systems with a single input polarization state of light, they are simpler to acquire and to calculate than more advanced PS-OCT-based information. However, these advanced metrics, including local retardation^22^ and optic axis^29^, could also be highly informative for longitudinal wound-healing studies and a more thorough assessment of collagen reorganization. We also envision our approach will potentially be useful in validating even simpler OCT-based measurements such as tissue attenuation coefficient^30^, a non-polarization-based metric for tissue characterization which could eliminate the need for polarization-diverse detection hardware.

The most significant limitation in the clinical interpretation of this work was the usage of porcine skin samples ex vivo. While porcine skin is accepted to be a better match for human skin than that of other research animals (such as rodents) in terms of some aspects of its architecture and immunological behavior^26^, there remain differences in anatomy and healing properties, making it an imperfect surrogate sample. Further, under our experimental conditions, the ex vivo tissue lacked any perfusion. We expect that the burn times and temperatures used in this study would produce different severities if replicated in vivo, with heat dissipation via blood flow greatly reducing overall severity. However, in the scope of this work, an ex vivo environment provided an optimal environment for highly controllable burn induction. Since the goal of our preliminary study was only to evaluate the ability of DOP maps to quantitatively determine the depth extent of thermal damage to collagen in burned samples, using samples with regions of uniform burn depth, unimpacted by potentially differing levels of perfusion, produced results that were easy to interpret and average across samples and experiments for robust evaluation. Although the severity and uniformity of burn wounds would differ in vivo, we nonetheless expect DOP to remain as strong of a metric in revealing underlying burn wound structure, making our results relevant regardless of the tissue perfusion status. Future work will focus on addressing each of these limitations with experiments in human skin in vivo. Blood flow in tissue also presents an interesting additional form of contrast to be explored by OCT angiography^30^ to provide assessment of local perfusion in injured areas and potentially augment PS-OCT measurements. One environmental parameter not fully explored in this work was how the temperature of the block used to induce the burn wound may impact polarimetric results. While it is possible that the optical properties of the burned tissue may differ depending on temperature of burn induction, such as an increased optical attenuation coefficient in charred tissue, we predict that the polarimetric properties of the burned tissue, namely the strong decrease in DOP, will continue to produce strong contrast. We have verified over a small additional temperature range (110 °C to 170 °C) that we are still able to achieve accurate histological results (Supplementary Fig. S3), but further work is necessary to confirm that burns induced at much higher temperatures are still able to be accurately assessed with our methods.

A fundamental limitation of OCT usage for many dermatological clinical applications is limited scan area. While OCT is still able to image far larger volumes than conventional microscopy techniques in far shorter time periods, many burn patients have injuries that cover a substantial proportion of total body surface area, rendering typical OCT fields of view (on the order of a few cubic centimeters) inadequate for full patient assessment in a reasonable amount of time. Nonetheless, our PS-OCT-based methods might be effectively combined with faster and higher-throughput tools such as visual assessment and digital photography, where areas of unclear severity could be spot-checked with a hand-held OCT probe. In the long run, the speed and range of OCT imaging continue to increase with fundamental advances such as ultrafast laser sources^31^ and circular ranging^32^, which promise to enable substantially larger-scale PS-OCT imaging in the future. Another potential limitation of OCT is its penetration depth; while  1-1.5mm is enough to assess the dermis at many anatomical locations, OCT sources at longer wavelengths (1.7 micron) could provide even deeper characterization of skin^33^.

Fundamentally, DOP assesses the amount of randomness in measured polarization states throughout a small ROI. The high DOP observed throughout injured dermis following burn induction, compared to the low DOP at comparable sites in healthy skin, is a direct structural result of this particular pathology, and lends itself to quantitative analysis. However, challenges remain in accurate analysis and interpretation of DOP. In these experiments, we applied an SNR correction, outlined in the Appendix, similar to a previously developed correction for DOP Uniformity^34^. Since DOP measurements are sensitive to additive noise in each polarization channel, this SNR correction helped produce a DOP signal that diminished less with depth than the uncorrected signal (Supplementary Fig. S4). Using the uncorrected signal resulted, for a given threshold, in either the overestimation of burn depth for shorter duration burns, or the underestimation of burn depth for longer duration burns, with respect to histological results. These SNR-uncorrected data provided similar results in burn depth estimation to those determined from maps of tissue birefringence. More complicated methods have been previously developed to apply a noise correction to local phase retardation calculations using maximum a posteriori estimation^35^. Especially for applications assessing the polarimetric signal relatively deep into a highly scattering medium, the simplicity of our methods bolsters the usage of DOP over birefringence or cumulative retardation to determine accurate collagen injury depth.

Since DOP is the measurement of randomness throughout an ROI, it depends in turn on the size of the ROI itself. We observed a reduction in DOP with increasing size of the filtering kernel used to calculate DOP, as well as some difference between each of the two input polarization states used to calculate DOP (Supplementary Fig. S5), though this disparity varied between the two Datasets, which were acquired three months apart. In general, we found that one input state measured consistently lower DOP (Supplementary Fig. S6)^36^; however, the lower DOP channel could still be used to achieve an accuracy comparable to the other input state, and thus the histologically-derived results, by altering the threshold at which the boundary between damaged and healthy collagen was defined. Each of these considerations motivates a full characterization of the PS-OCT system used in any measurements of burn injury depth. Further, simple calibration measurements using tissue-mimicking birefringence phantoms^37^ could ensure consistency between measurements and analysis protocols to report accurate burn depth measurements without unconsidered bias from the PS-OCT system.

In the analysis of both PS-OCT-based data and histological data, we used image processing and thresholding at empirically-determined values to estimate burn depth. Although the PLM results provided the clearest binary signal in ground-truth burn depth evaluation, future studies would benefit from the addition of more sophisticated immunostaining for molecular markers of thermal damage. Our methods perform a structural assessment of tissue rather than a functional one, and thus the presence or absence of intact collagen using histological analysis validates the DOP behavior observed with PS-OCT. However, more functionally probing stains could help to fill the unmet gap in correlating the specific depth extent of collagen damage to functional assessments and healing in patients, which is currently a limitation to direct clinical translation of our approach. It could also be helpful to correlate observations to theoretical predictions such as through the Arrhenius damage integral^6^, but longitudinal studies will be most useful for refining and validating DOP metrics for injury assessment and for determining how these metrics anticipate functional outcomes.

Automated, comprehensive detection of hair follicles and other adnexal structures such as sweat glands within OCT images of burn wounds remains an interesting challenge. Determining locations of mature follicles contextualizes collagen injury depth: although dermal and epidermal thickness can vary throughout the body, follicle location helps to differentiate relatively deeper dermal areas. Future work could also confirm whether follicular damage occurs in parallel with collagen damage in human skin, as previously reported in rat skin^27^. Although we have also confirmed this effect in our tissue samples via a preliminary histological analysis based on the same structural criteria (Supplementary Fig. S2), the functional stains discussed above could also be useful in this validation, and analysis in vivo would provide even more definitive results. Lower content of sweat glands in porcine skin further supports using human skin samples for experiments both in vivo and ex vivo if possible, and especially for studies examining regeneration or wound healing. Human sweat glands visualized using OCT have been previously reported to have a unique, spirally-shaped structure^38^, and their relevance as adnexal structures bearing stem cells merits further investigation to automate their identification^39^. Our basic approach to identify hair follicles and hair shafts from OCT images could be significantly improved by the application of more sophisticated methods including machine learning^40^ or advanced parametric image processing, which are both compatible with insertion into our existing image processing pipeline, and would be further extendable to identify other unique and relevant structures such as sweat glands. Although such methods were beyond the scope of this current work, which only sought to demonstrate the feasibility of adding follicle information to complement PS-OCT analysis, we hope that they will be the subject of future work for this and other dermatological applications.

## Methods

### Porcine tissue samples

A 100 $\text{cm}^2$ sample of skin from the flank region of an adult pig was acquired post mortem from either from a commercial abbatoir vendor (Dataset 1: Lampire Biological Laboratories, PA) or from a collaborator at Massachusetts General Hospital (Dataset 2). In the former case, the tissue was received forty-eight hours post-harvest; in the latter case, the tissue was received immediately after harvesting. In both cases, the tissue was maintained on ice and hydration was maintained with saline throughout the preparation and imaging procedures, a period of approximately two hours. As part of the tissue preparation procedure, hair was removed superficially from the porcine skin with a razor. While the presence of hair is not in itself a limitation to OCT imaging or PS-OCT processing, its removal was desirable to make the burn induction as uniform as possible. Burns were induced in the tissue samples at precise intervals of 1.5s, 3s, 5s, 8s, 15s, 30s, and 60s to encompass a large range (superficial to deep partial or full thickness) of wound severities. Unless otherwise noted, burns were induced at 150 $^{\circ }$C using a brass block with a surface area of 0.5 cm $\times$ 1 cm. The brass block was fitted to a soldering iron and continuously heated at 150 $^{\circ }$C to ensure the element did not significantly cool over time, a limiting factor for deep burn induction in previous studies^41^. After all imaging had concluded, tissue was fixed in 10% formalin for two weeks, then paraffin embedded and sectioned as 5-micron cross sections. Sections were either processed with Hematoxylin and Eosin (H&E) staining or left unstained. Four cross sections (2 stained, 2 unstained) were taken at regular intervals from each 0.5 cm$^2$ burned region. Within each Dataset, burns of each duration were induced in three different areas. In total, this provided $N=3$ PS-OCT imaged volumes with corresponding surface area of 0.5 cm$^2$, $N=6$ H&E-stained cross sections, and $N=6$ unstained cross sections, for each Dataset. H&E-stained sections were scanned and recorded as high-resolution digital pathology images (Nanozoomer, Hamamatsu). Unstained slides were imaged with polarized light microscopy (PLM) using a custom microscope setup featuring crossed circular polarizers for improved characterization of collagen fiber content.

### OCT imaging

The PS-OCT system used to acquire data in these experiments is described in detail elsewhere^22^. Briefly, the system used a wavelength-swept laser centered at 1290 nm with a bandwidth of 115 nm, an A-line rate of 100 kHz, and an axial resolution of 9.4 $\mu$m in air (or  13 $\mu$m in tissue). In the sample arm, an electrooptic modulator was used to alternate between circular and linear polarization states for adjacent A-lines. In the reference arm, an acoustooptic modulator was used to remove depth degeneracy. A galvanometer scanner (CambridgeTech/Novanta) was used to translate the beam, focused by a 5X scanning objective lens (Thorlabs, LSM03), across the sample with a lateral resolution of 30 $\mu$m (full-width half maximum, FWHM). Each volume was acquired over a 1.2 cm $\times$ 0.3 cm area at Nyquist sampling. The interference signal was recorded with polarization-diverse and balanced detection in two output polarization channels. All data were analyzed in MATLAB. All OCT intensity data are displayed on a logarithmic scale as the signal-to-noise ratio (SNR).

### Signal and image processing

#### PS-OCT

Following conventional tomogram reconstruction and the calculation of the Stokes vectors (*Q*, *U*, *V*) and intensity (*I*) from the complex tomogram, the DOP, represented here by $\varphi$, was calculated as

1 $$\begin{aligned} \varphi _p = \frac{\sqrt{\langle Q_p\rangle ^2 + \langle U_p\rangle ^2 + \langle V_p\rangle ^2}}{\langle I_p\rangle } \end{aligned}$$

where *p* denotes the input polarization state, and $\langle ...\rangle$ denotes ensemble averaging, or spatial averaging over an area large enough to capture the signal from different resolution volumes (speckle), implemented by means of a convolution. Gaussian filtering was applied over lateral (in-plane) and axial kernels of 3 and 2 pixels FWHM (35 and 9 $\upmu$m, respectively). A correction for DOP based on the reduction of signal-to-noise ratio (SNR) with depth was applied using methods similar to Ref.^34^ (see SNR Correction and Supplementary Fig. S3 online). Although the two-input-state PS-OCT system afforded the opportunity to average the DOP across both input states, only one input state was used in burn depth estimation to demonstrate the feasibility of performing these experiments with a simpler single-input-state system. Differences in behavior between DOP calculated from each input state are examined in Supplementary Fig. S5.

Although the focus of this work was to investigate DOP as a predictor of burn depth, tissue birefringence was also quantified to provide supplementary contrast and context to the DOP maps. For birefringence calculations, data were reconstructed with spectral binning^22^, using 9 overlapping bins, each with a width of 1/5 of the full spectrum, and the same filtering parameters described above used for the DOP calculations. An axial offset of 6 pixels (28 $\upmu$m) was used to derive depth-resolved birefringence from the two input polarization states. The birefringence is given in degrees/$\upmu$m, corresponding to the amount of retardation per sample path.

An automated image processing pipeline used SNR-corrected DOP maps generated using PS-OCT processing to estimate burn wound injury depth. First, the surface of the tomogram was detected automatically. With a DOP threshold set to 0.95, the boundary beneath the surface at which the DOP fell below this value was identified, corresponding to healthy dermis and the return of a birefringent collagen signal. In unburned areas, this analysis resulted in non-zero burn depth estimates due to the lack of birefringence (and resulting high DOP) throughout the epidermis. The apparent depth extent of the collagen injury was determined with respect to the sample surface and mapped volumetrically *en face*. Depths in pixel units were converted to physical distances based on the ranging depth of the PS-OCT system (6 mm) and an estimated group refractive index for the dermis, composed primarily of water and collagen, of 1.41^42^. While it is possible that the tissue refractive index changed as a result of collagen denaturation and dermal dehydration, it is unlikely that this change would be of sufficient magnitude to substantially impact our results. Regions of interest (ROIs) spanning a representative sector of each burned volume, corresponding to a surface area of at least 3 mm $\times$ 2.5 mm, were used to calculate the mean collagen injury depth throughout the volume. These mean values were averaged again across each of the three volumes acquired for each Dataset, and used to calculate corresponding standard deviations.

#### Histology

Polarized light microscopy (PLM) imaging of unstained tissue sections was used as a ground truth method to determine the depth-extent of collagen injury in burned tissue samples. For each cross-sectional PLM image, the mean background signal was acquired and used to correct for any variation in light intensity within or across samples. The tissue surface was automatically extracted, as was the depth below the surface at which a sufficient birefringence signal was detected, corresponding to the presence of a healthy dermal collagen matrix. The mean injury depth was calculated from these two curves, then further averaged across the six cross sections for each burn duration obtained for each Dataset. Standard deviation was determined across these six independent measurements for each Dataset.

The gelatinization zone in corresponding H&E-stained cross sections was used as an additional metric of collagen injury depth extent. In high-resolution digital pathology images, analogous image processing methods were used to detect the tissue surface and the boundary of the gelatinization zone based on Red-Green-Blue (RGB) thresholding, using threshold values optimized to match corresponding PLM results. As with the PLM results, the mean injury depth was calculated for each of the $N=6$ cross sections for each Dataset, averaged for each burn duration, and used to calculate standard deviation.

#### Follicle identification

From OCT intensity data, a basic thresholding and image processing scheme was used to identify adnexal structures (e.g. hair follicles) as small, elliptically-shaped areas of relatively low intensity signal. A 2-D Wiener filter was applied to each cross-sectional image within the volume, and the tissue surface was identified, as was the point below the surface at which the intensity reached the noise floor. Tomograms were depth-compensated for attenuation, and resulting logarithmic intensity cross sections were thresholded to a value 5 dB above the noise floor. Identified low-signal areas were selected using built-in MATLAB function based on their shape (imclose; ’disk’) and size (bwareaopen). Identified regions with area above an empirically-determined maximum value were ignored as shadows of superficial hair shafts. Remaining areas were classified as hair follicles (Fig. 4b); *en face* hair follicle maps were further smoothed with bwareaopen to highlight adjacent frames showing follicles evolving in depth.

## Supplementary Information

Supplementary Information.

## Data Availability

Data underlying the results presented are not publicly available at this time but may be obtained from the corresponding author upon reasonable request.

## References

[1] Burn Incidence Fact Sheet. (2016).

[2] Pape SA, Skouras CA, Byrne PO (2001). An audit of the use of laser Doppler imaging (LDI) in the assessment of burns of intermediate depth. Burns.

[3] Heimbach D, Engrav L, Grube B, Marvin J (1992). Burn depth: A review. World Journal of Surgery.

[4] Singh V, Devgan L, Bhat S, Milner SM (2007). The Pathogenesis of Burn Wound Conversion. Annals of Plastic Surgery.

[5] Kaiser M, Yafi A, Cinat M, Choi B, Durkin AJ (2011). Noninvasive assessment of burn wound severity using optical technology: A review of current and future modalities. Burns.

[6] Orgill DP, Solari MG, Barlow MS, O’Connor NE (1998). A finite-element model predicts thermal damage in cutaneous contact burns. J. Burn Care Rehabil..

[7] Gentile P, Scioli MG, Bielli A, Orlandi A, Cervelli V (2017). Stem cells from human hair follicles: First mechanical isolation for immediate autologous clinical use in androgenetic alopecia and hair loss. Stem Cell Invest..

[8] Wolman M, Gillman T (1972). A polarized light study of collagen in dermal wound healing. Br. J. Exp. Pathol..

[9] Pearce, J. A., Thomsen, S. L., Vijverberg, H. & McMurray, T. J. Kinetics for birefringence changes in thermally coagulated rat skin collagen. In *OE/LASE’93: Optics, Electro-Optics, & Laser Applications in Science & Engineering* (eds Anderson, R. R., Bass, L. S., Shapshay, S. M., White, J. V. & White, R. A.) 180 (Los Angeles, CA, 1993). 10.1117/12.147029.

[10] Huang D (1991). Optical coherence tomography. Science.

[11] Hee MR, Huang D, Swanson EA, Fujimoto JG (1992). Polarization-sensitive low-coherence reflectometer for birefringence characterization and ranging. JOSA B.

[12] de Boer JF, Milner TE, van Gemert MJC, Nelson JS (1997). Two-dimensional birefringence imaging in biological tissue by polarization-sensitive optical coherence tomography. Opt. Lett..

[13] de Boer JF, Hitzenberger CK, Yasuno Y (2017). Polarization sensitive optical coherence tomography: A review [Invited]. Biomed. Opt. Express.

[14] Nguyen JQ (2020). Early visualization of skin burn severity using a topically applied dye-loaded liquid bandage. Sci. Rep..

[15] Calzavara-Pinton P, Longo C, Venturini M, Sala R, Pellacani G (2008). Reflectance confocal microscopy for in vivo skin imaging. Photochem. Photobiol..

[16] Rangaraju LP (2019). Classification of burn injury using Raman spectroscopy and optical coherence tomography: An ex-vivo study on porcine skin. Burns.

[17] Oltulu P, Ince B, Kokbudak N, Findik S, Kilinc F (2018). Measurement of epidermis, dermis, and total skin thicknesses from six different body regions with a new ethical histometric technique. Turk. J. Plast. Surg..

[18] Park BH, Saxer C, Srinivas SM, Nelson JS, de Boer JF (2001). In vivo burn depth determination by high-speed fiber-based polarization sensitive optical coherence tomography. J. Biomed. Opt..

[19] Kim KH (2012). In vivo imaging of human burn injuries with polarization-sensitive optical coherence tomography. J. Biomed. Opt..

[20] Pierce MC, Sheridan RL, Hyle Park B, Cense B, de Boer JF (2004). Collagen denaturation can be quantified in burned human skin using polarization-sensitive optical coherence tomography. Burns.

[21] Jiao S, Wurong Y, Stoica G, Wang LV (2003). Contrast mechanisms in polarization-sensitive Mueller-matrix optical coherence tomography and application in burn imaging. Appl. Opt..

[22] Villiger M (2013). Spectral binning for mitigation of polarization mode dispersion artifacts in catheter-based optical frequency domain imaging. Opt. Express.

[23] Lo WC (2016). Longitudinal, 3D imaging of collagen remodeling in murine hypertrophic scars in vivo using polarization-sensitive optical frequency domain imaging. J. Investig. Dermatol..

[24] Golberg A (2016). Preventing scars after injury with partial irreversible electroporation. J. Investig. Dermatol..

[25] Karim AS (2019). Discordance between histologic and visual assessment of tissue viability in excised burn wound tissue: Visual-molecular discordance in burn tissues. Wound Repair Regener..

[26] Summerfield A, Meurens F, Ricklin ME (2015). The immunology of the porcine skin and its value as a model for human skin. Mol. Immunol..

[27] Meyerholz DK, Piester TL, Sokolich JC, Zamba GKD, Light TD (2009). Morphological parameters for assessment of burn severity in an acute burn injury rat model. Int. J. Exp. Pathol..

[28] Park BH, Pierce MC, Cense B, de Boer JF (2004). Jones matrix analysis for a polarization-sensitive optical coherence tomography system using fiber-optic components. Opt. Lett..

[29] Li Q, Sampson DD, Villiger M (2020). In vivo imaging of the depth-resolved optic axis of birefringence in human skin. Opt. Lett..

[30] Gong P (2013). Assessment of human burn scars with optical coherence tomography by imaging the attenuation coefficient of tissue after vascular masking. J. Biomed. Opt..

[31] Klein T, Huber R (2017). High-speed OCT light sources and systems [Invited]. Biomed. Opt. Express.

[32] Siddiqui M (2018). High-speed optical coherence tomography by circular interferometric ranging. Nature Photonics.

[33] Li Y (2021). 1.7-Micron optical coherence tomography angiography for characterization of skin lesions: A feasibility study. IEEE Trans. Med. Imaging.

[34] Makita S, Hong Y-J, Miura M, Yasuno Y (2014). Degree of polarization uniformity with high noise immunity using polarization-sensitive optical coherence tomography. Opt. Lett..

[35] Kasaragod D, Makita S, Hong Y-J, Yasuno Y (2017). Noise stochastic corrected maximum a posteriori estimator for birefringence imaging using polarization-sensitive optical coherence tomography. Biomed. Opt. Express.

[36] Lippok N, Villiger M, Bouma BE (2015). Degree of polarization (uniformity) and depolarization index: Unambiguous depolarization contrast for optical coherence tomography. Opt. Lett..

[37] Liu X (2017). Tissue-like phantoms for quantitative birefringence imaging. Biomed. Opt. Express.

[38] Tripathi SR, Miyata E, Ishai PB, Kawase K (2015). Morphology of human sweat ducts observed by optical coherence tomography and their frequency of resonance in the terahertz frequency region. Sci. Rep..

[39] Gong L, Xu X-G, Li Y-H (2018). Embryonic-like regenerative phenomenon: Wound-induced hair follicle neogenesis. Regener. Med..

[40] Urban G (2021). Combining deep learning with optical coherence tomography imaging to determine scalp hair and follicle counts. Lasers Surg. Med..

[41] Srinivas SM (2004). Determination of burn depth by polarization-sensitive optical coherence tomography. J. Biomed. Opt..

[42] Tearney GJ (1995). Determination of the refractive index of highly scattering human tissue by optical coherence tomography. Opt. Lett..

