# Supplementary material for: Measuring collagen injury depth for burn severity determination using polarization sensitive optical coherence tomography
Source: Sci Rep. 2022 Jun 21;12:10479. doi: 10.1038/s41598-022-14326-3 (PMC9213509; doi:10.1038/s41598-022-14326-3)
Supplement: Supplementary file 1 — Supplementary Information. [file 41598_2022_14326_MOESM1_ESM.pdf]

Measuring collagen injury depth for burn severity determination using polarization sensitive optical coherence tomography: Supplementary information

Taylor M. Cannon[1, 2, *], Néstor Uribe-Patarroyo[2], Martin Villiger[2], and Brett E. Bouma[1,2]

[1]Institute for Medical Engineering and Science, Massachusetts Institute of Technology, Cambridge, MA, 02142, USA
[2]Wellman Center for Photomedicine, Massachusetts General Hospital, Boston, MA, 02114, USA
[*]cannont@mit.edu

1 SNR Correction

DOP is calculated for a given input polarization state from complex data containing both signal, S, and noise, N. For simplicity, in this section, we do not specify the input polarization state (p), but apply the same SNR correction equivalently to any general input polarization state. We denote the full OCT signal as F, where $F = S + N$. N follows a zero-mean complex circular Gaussian distribution.[1] S is also given by a zero-mean complex circular Gaussian distribution, but has different temporal dynamics, and we assume that S and N are uncorrelated.[2] Therefore, $\langle SN\rangle = \langle S\rangle\langle N\rangle$, where $\langle ...\rangle$ denotes an ensemble average, or spatial averaging over an area large enough to capture the signal from different resolution volumes (speckle), implemented by means of a convolution. For each of the two detection channels, denoted x and y, we have $F_x = S_x + N_x$, and $F_y = S_y + N_y$. The intensity signal combined across the two detection channels, $\langle I\rangle$, is given by,

$$\langle I\rangle = \left\langle |F_x|^2\right\rangle + \left\langle |F_y|^2\right\rangle = \left\langle |S_x|^2\right\rangle + \left\langle |N_x|^2\right\rangle + \left\langle |S_y|^2\right\rangle + \left\langle |N_y|^2\right\rangle + 2\mathrm{Re}\{S_x^* N_x\} + 2\mathrm{Re}\{S_y^* N_y\}. \quad (1)$$

Since $\left\langle N_{x,y}\right\rangle = 0$, this simplifies to

$$\langle I\rangle = \left\langle |S_x|^2\right\rangle + \left\langle |N_x|^2\right\rangle + \left\langle |S_y|^2\right\rangle + \left\langle |N_y|^2\right\rangle = \langle I_S\rangle + \langle I_N\rangle, \quad (2)$$

where I_S and I_N are the intensities of the signal and noise summed over the two detection channels, respectively.

To calculate DOP, we further derive the signal and noise components of the Stokes vectors Q, U, and V. Similarly to $\langle I\rangle$, $\langle Q\rangle$ is given by

$$\langle Q\rangle = \left\langle |F_x|^2\right\rangle - \left\langle |F_y|^2\right\rangle = \left\langle |S_x|^2\right\rangle + \left\langle |N_x|^2\right\rangle - \left\langle |S_y|^2\right\rangle - \left\langle |N_y|^2\right\rangle = \langle Q_S\rangle + \langle Q_N\rangle, \quad (3)$$

after the terms incorporating $\left\langle N_{x,y}\right\rangle = 0$ are removed. We note that if the noise is the same in both detection channels, then $\langle Q_N\rangle = 0$, and $\langle Q\rangle = \langle Q_S\rangle$. Ideally, the noise floor should be identical for each detection channel in OCT systems using balanced detection. In practice, imperfect and spectrally-dependent balancing may lead to disparate impact of noise between x and y channels, thus making it necessary to account for Q_N.

For the remaining Stokes vectors,

$$\langle U\rangle = \left\langle 2\mathrm{Re}\{F_x F_y^*\}\right\rangle = \left\langle F_x F_y^* + F_x^* F_y\right\rangle = \left\langle (S_x + N_x)(S_y^* + N_y^*)\right\rangle + \left\langle (S_y + N_y)(S_x^* + N_x^*)\right\rangle. \quad (4)$$

Using $\left\langle N_{x,y}\right\rangle = 0$, this simplifies to

$$\langle U\rangle = \left\langle S_x S_y^*\right\rangle + \left\langle S_y S_x^*\right\rangle = \langle U_S\rangle. \quad (5)$$

and similarly,

$$\langle V\rangle = \left\langle -2\mathrm{Im}\{F_x F_y^*\}\right\rangle = \left\langle S_x S_y^*\right\rangle - \left\langle S_y S_x^*\right\rangle = \langle V_S\rangle. \quad (6)$$

In calculating DOP for a given input polarization state, represented here by φ, the following contribution of noise is included:

$$\varphi^2 = \frac{\langle Q\rangle^2 + \langle U\rangle^2 + \langle V\rangle^2}{\langle I\rangle^2} = \frac{\langle Q_S\rangle^2 + \langle Q_N\rangle^2 + 2\langle Q_S\rangle\langle Q_N\rangle + \langle U_S\rangle^2 + \langle V_S\rangle^2}{\langle I_S\rangle^2 + \langle I_N\rangle^2 + 2\langle I_S\rangle\langle I_N\rangle} \quad (7)$$

$$\varphi^2 = \frac{\langle Q_S\rangle^2 + \langle U_S\rangle^2 + \langle V_S\rangle^2}{\langle I_S\rangle^2} \frac{1}{1 + \frac{\langle I_N\rangle^2}{\langle I_S\rangle^2} + 2\frac{\langle I_N\rangle}{\langle I_S\rangle}} + \frac{\langle Q_N\rangle^2 + 2\langle Q_S\rangle\langle Q_N\rangle}{\langle I_S\rangle^2(1 + \frac{\langle I_N\rangle^2}{\langle I_S\rangle^2} + 2\frac{\langle I_N\rangle}{\langle I_S\rangle})}, \tag{8}$$

where the first term corresponds to the desired DOP of the signal without noise, φ_S. This may be written in terms of the signal-to-noise ratio (SNR), here defined as $R_x = \frac{\langle S_x|^2\rangle}{\langle |N_x|^2\rangle}$ and similarly for y polarization detection channel,

$$\varphi^2 = \left(\varphi_S^2 + \frac{\langle Q_N\rangle^2 + 2\langle Q_S\rangle\langle Q_N\rangle}{\langle I_S\rangle^2}\right) \frac{1}{1 + 2R_{xy}^{-1} + R_{xy}^{-2}}, \tag{9}$$

where $R_{xy} = \frac{\langle I_S\rangle}{\langle I_N\rangle}$. DOP without the contribution of noise, φ_S, may be solved for as

$$\varphi_S = \sqrt{\varphi^2(1 + 2R_{xy}^{-1} + R_{xy}^{-2}) - \beta}, \tag{10}$$

where $\beta = \frac{\langle Q_N\rangle^2 + 2\langle Q_S\rangle\langle Q_N\rangle}{\langle I_S\rangle^2}$. We define the SNR ($R$) factors $r = 1 + R$ and $q = 1 + R^{-1}$ such that $\langle |N|^2\rangle = \frac{\langle |F|^2\rangle}{1+R} = \frac{\langle |F|\rangle^2}{r}$ and $\langle |S|^2\rangle = \frac{\langle |F|^2\rangle}{1+R^{-1}} = \frac{\langle |F|^2\rangle}{q}$. The β term may now be written in terms of the tomogram measured for each input state and the noise floor characterized for the PS-OCT system:

$$\beta = \frac{\langle |F_x|^2\rangle \left(r_x^{-2} + 2r_x^{-1}q_x^{-1}\right) + \langle |F_y|^2\rangle \left(r_y^{-2} + 2r_y^{-1}q_y^{-1}\right) - 2\langle |F_x|^2\rangle\langle |F_y|^2\rangle \left(r_x^{-1}r_y^{-1} + q_x^{-1}r_y^{-1} + q_y^{-1}r_x^{-1}\right)}{\langle |F_x|^2\rangle q_x^{-2} + \langle |F_y|^2\rangle q_y^{-2} + 2\langle |F_x|^2\rangle\langle |F_y|^2\rangle (q_x q_y)^{-2}}. \tag{11}$$

2 Supplementary figures

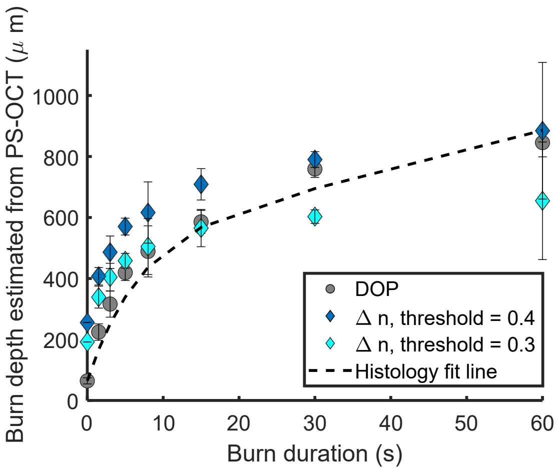

Figure S1. In addition to DOP, birefringence (Δ n) was mapped throughout each porcine tissue sample, and burned regions were found to have low Δn. Results using a birefringence-based threshold value to determine the depth extent of collagen injury damage from the PS-OCT data (e.g., an increase in birefringence indicating the border between damaged and healthy dermis) were similar to those obtained using DOP-based contrast without SNR correction, though less representative of histologically-determined burn depth than SNR-corrected DOP. Two representative threshold levels are shown (Δ n = 0.3 degrees (deg)/μm, 0.4 deg/μm). Data shown were averaged across each Dataset.

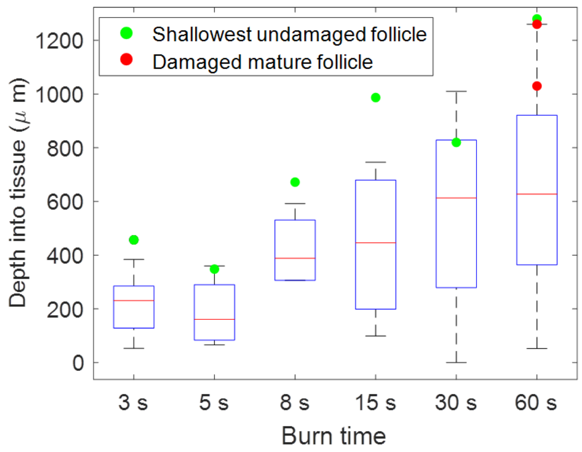

Figure S2. Hair follicles and cross-sections of hair shafts were identified in all Hematoxylin and Eosin (H&E)-stained tissue sections in which burns of 3 s or greater duration had been induced, and evaluated for signs of thermal damage based on the criteria detailed by Meyerholz *et al.*[3] Depths of thermally-damaged hair follicles were recorded and the ranges of these depths were summarized as a box plot for sections of each burn duration. In general, the depths at which thermally-damaged follicles were identified increased with increasing burn duration, as did the depths of the shallowest undamaged follicles. Two damaged mature follicles were identified in tissues with burns induced for 60 s. In the box plots, the red line indicates the median depth of damaged follicles, the borders of the blue box indicate data at the 25th and 75th percentiles, and whiskers indicate the most extreme outliers for each group.

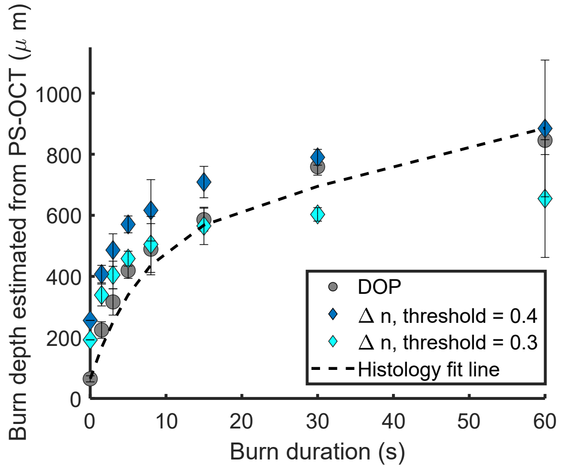

Figure S3. In addition to DOP, birefringence (δ n) was mapped throughout each porcine tissue sample, and burned regions were found to have low δ n. Results using a birefringence-based threshold value to determine the depth extent of collagen injury damage from the PS-OCT data (e.g., an increase in birefringence indicating the border between damaged and healthy dermis) were similar to those obtained using DOP-based contrast without SNR correction, though less representative of histologically-determined burn depth than SNR-corrected DOP. Two representative threshold values are shown (δ n = 0.3 deg/μ m, δ n = 0.4 deg/μ m). Data shown were averaged across each Dataset.

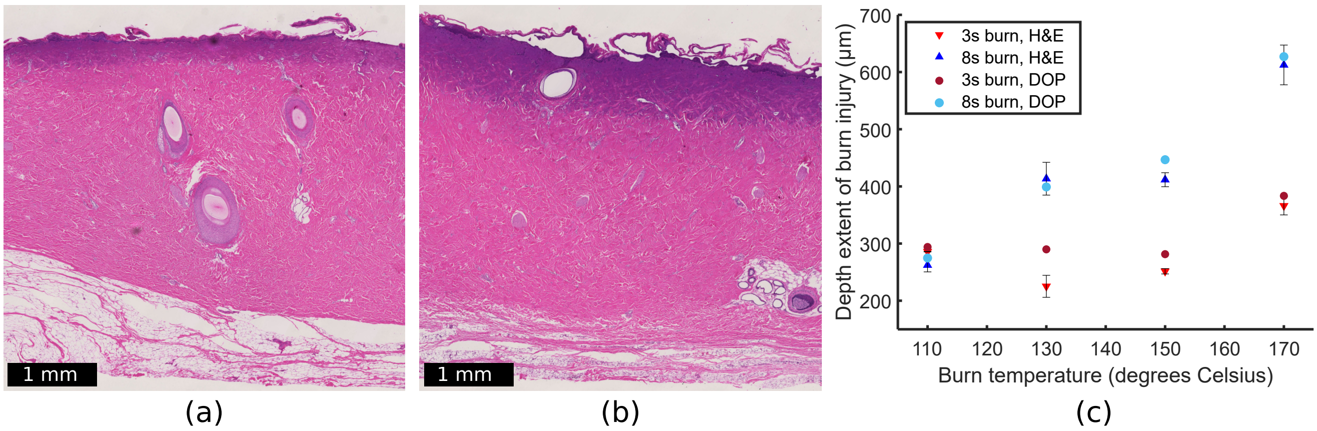

Figure S4. Representative histological cross sections for burns induced at 110 degrees Celsius (a) and 170 degrees Celsius (b) for a duration of eight seconds. Burn severity was found, as expected, to depend on both temperature and duration of induction (c). Multi-temperature data were only acquired for Dataset 2.

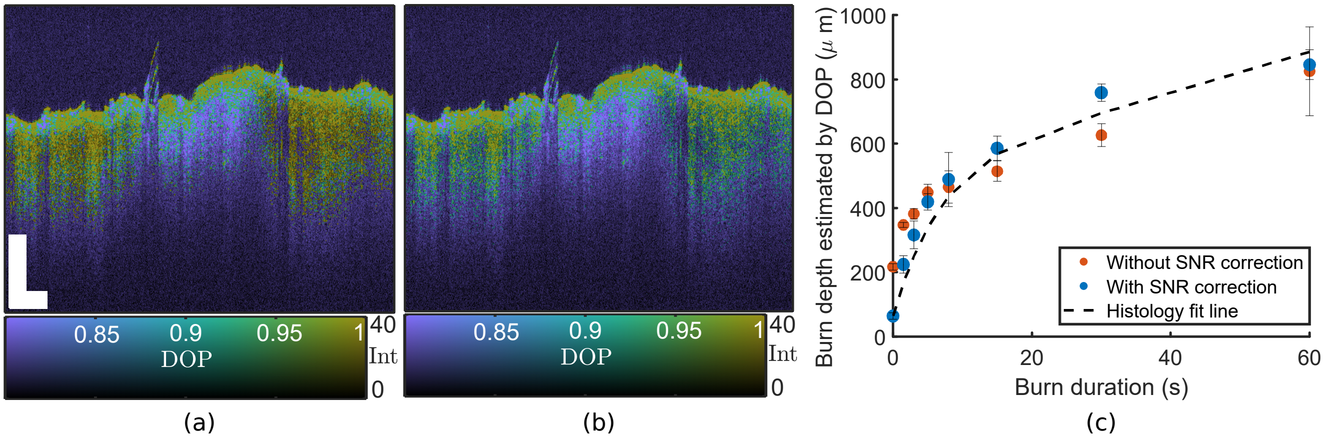

Figure S5. A signal-to-noise ratio (SNR) correction was applied to compensate for the noise present in each output polarization channel of the PS-OCT system. Our SNR-corrected DOP maps (a) maintained higher DOP values deeper into tissue compared to SNR-uncorrected maps (b). The cross-section displayed features a 15-s burn region on the left side, intact tissue in the middle, and a 30-s burn region on the right side. Using an SNR correction produced DOP-based results closer to the desired histologically validated results in assessing collagen injury depth (c), whereas without SNR correction, collagen injury depth was overestimated at short burn durations. Data in (c) were averaged across Datasets 1 and 2.

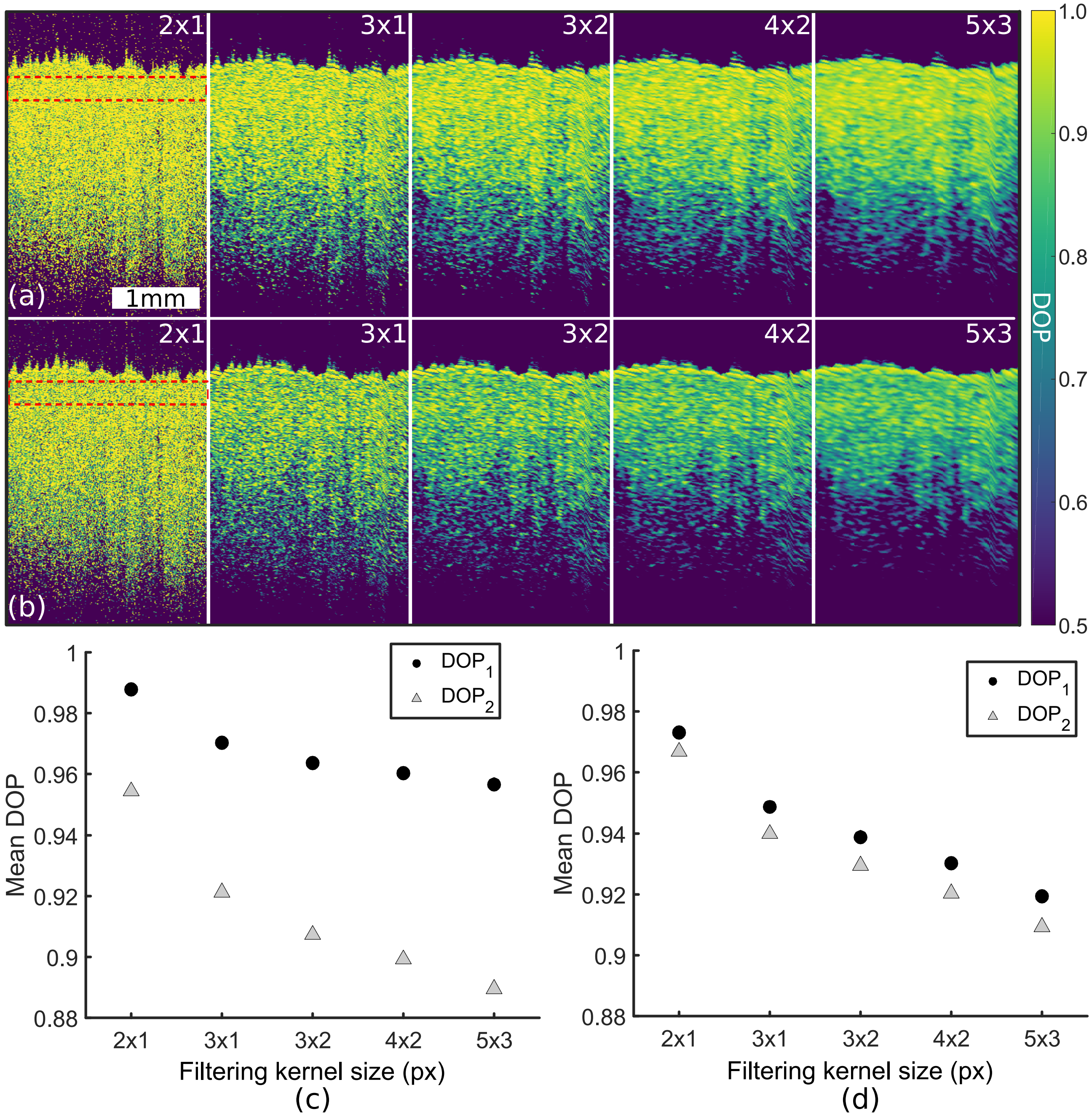

Figure S6. A representative cross sectional DOP map of a 30s-burn region is shown with different filtering kernels applied to calculate DOP for each input polarization state (a, b). The size of the filtering kernel in pixels (depth dimension x lateral dimension) is noted in text on each frame. The DOP was averaged for each filtering case in a superficial ROI (red dashed box) and plotted for each input polarization state (DOP_1, DOP_2) for Datasets 1 (c) and 2 (d), for which the relative impact of filtering different between each polarization state is shown.

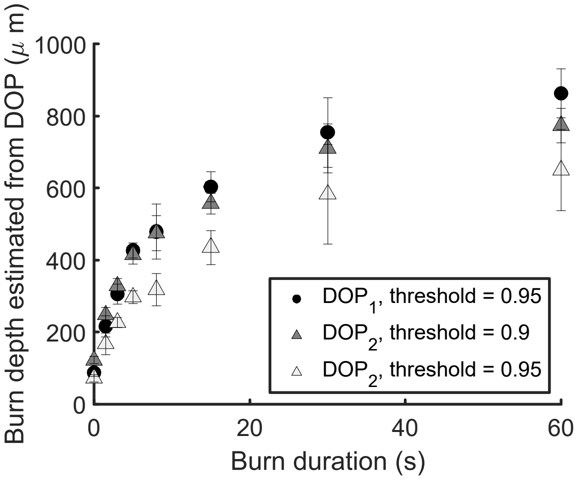

Figure S7. DOP was calculated for each input polarization state of the PS-OCT system. One input state produced consistently higher DOP, resulting in a difference in estimated burn depths. However, by lowering the cutoff threshold for "healthy" collagen detection in the lower DOP channel, similar results were obtained, demonstrating the feasibility of using a single input state PS-OCT system when appropriate calibrating methods are applied. Data shown were averaged across Datasets 1 and 2.

References

1. Makita, S., Kurokawa, K., Hong, Y.-J., Miura, M. & Yasuno, Y. Noise-immune complex correlation for optical coherence angiography based on standard and jones matrix optical coherence tomography. *Biomed. Opt. Express* **7**, 1525–1548, DOI: 10.1364/BOE.7.001525 (2016).
2. Uribe-Patarroyo, N., Post, A. L., Ruiz-Lopera, S., Faber, D. J. & Bouma, B. E. Noise and bias in optical coherence tomography intensity signal decorrelation. *OSA Continuum* **3**, 709–741, DOI: 10.1364/OSAC.385431 (2020).
3. Meyerholz, D. K., Piester, T. L., Sokolich, J. C., Zamba, G. K. D. & Light, T. D. Morphological parameters for assessment of burn severity in an acute burn injury rat model. *Int. J. Exp. Pathol.* **90**, 26–33, DOI: 10.1111/j.1365-2613.2008.00617.x (2009).
